# Quality midwifery care during the COVID-19 pandemic in Bangladesh: A focus group study with midwives, nurses, and midwifery educators

**DOI:** 10.18332/ejm/174234

**Published:** 2023-12-15

**Authors:** Noor I. Pappu, Jonna Holmedahl, Svava Gudjonsdottir, Kerstin Erlandsson, Ulrika Byrskog

**Affiliations:** 1 School of Health and Welfare, Dalarna University, Falun, Sweden

**Keywords:** Bangladesh, COVID-19 pandemic, focus group discussions, midwife profession, quality midwifery care

## Abstract

**INTRODUCTION:**

Bangladesh has made remarkable strides in the development of the midwifery profession. However, the COVID-19 pandemic has had profound effects on healthcare systems worldwide, including those related to reproductive, perinatal, and maternal health. Given the recent advancements in the midwifery field in Bangladesh, it is crucial to examine the pandemic's impact on existing barriers and the capacity of midwifery professionals to deliver high-quality care. The aim of this study is to describe the possibility of midwives being able to provide quality midwifery care in Bangladesh during the COVID-19 pandemic.

**METHODS:**

To gather insights, data were collected from July to October 2020 via four qualitative focus group discussions online; 23 actively practicing midwives, nurses specializing in midwifery care, and midwifery educators, participated. The data analysis employed reflexive thematic analysis.

**RESULTS:**

The COVID-19 crisis posed significant threats to women's safety and health, with lockdowns exacerbating gender inequalities in society. Midwives faced added challenges due to their relatively low professional status and increased workloads. Insufficient policy implementation further compromised midwives' safety. Fear of contracting the virus and working during their free time also raised concerns about the quality of care provided. Nevertheless, the pandemic provided opportunities for midwives to demonstrate their ability to deliver independent midwifery care in Bangladesh.

**CONCLUSIONS:**

The pandemic underscored the importance of creating respectful and dignified working conditions for midwives. It revealed that professional midwives can work independently when provided with the necessary space and a supportive work environment. This opens the door for the implementation of a midwifery-led care model. Further research is recommended to investigate the medical safety and efficacy of independent midwifery care in the context of Bangladesh.

## INTRODUCTION

The midwifery profession in Bangladesh has undergone significant development in recent years, brought on by global evidence of the strong links between skilled and professional midwifery care and improved maternal and neonatal health outcomes^1,2^. Due to the COVID-19 pandemic severely affecting healthcare systems worldwide^3^, it is vital to future practice that its influence on the newly established midwifery profession is examined.

Bangladesh, with a total population of over 162 million people^4^, has experienced sustained economic growth in recent decades, which has reduced poverty and allowed the country to reach the status of a lower middle-income country (LMIC) in 2015^5^. Since 2010, the Bangladeshi government has recognized midwifery as a profession as outlined by the International Confederation of Midwives (ICM). In collaboration with the World Health Organization, United Nations Population Fund, and other organizations, a steady development of professional midwifery education has been ensured alongside the development of the role and responsibilities of midwives in the country^6^. Professional midwives in Bangladesh have their own national organization – The Bangladesh Midwifery Society – and are registered and licensed under the Bangladesh Nursing and Midwifery Council since 2010^6^. The Directorate General of Nursing and Midwifery is guided by an ethical code and standard operating procedures in their work to govern the services delivered by licensed midwives^6^. Currently, midwifery care in Bangladesh is provided by registered midwives educated according to international standards, and nurse-midwives with a shorter post-basic training in midwifery^6^.

Bangladesh has thus taken significant steps in developing the midwifery profession during a short period of time^6^. Even so, many barriers to providing quality maternal and newborn care still exist^7^. These include vulnerability due to gender inequality, lack of professional recognition, medical hierarchy, low level of autonomy, low and/or irregular salaries, insecurity due to lack of housing and transportation, and a lack of recreation^8,9^. Furthermore, a lack of logistic support including equipment, essential drugs, and laboratory requirements, as well as a lack of healthcare personnel, heavy workloads, and overcrowding in hospitals are existing factors that contribute to a reduced midwifery quality of care in Bangladesh^9,10^. Midwives need support to achieve and maintain self-confidence through continuous development in education, professionalism, and economic compensation in order to enable quality midwifery care^8^.

December 2019 saw the outbreak of a coronavirus in Wuhan, China, resulting in the infectious disease COVID-19 spreading rapidly around the world causing a pandemic^11^. In early 2023, almost 757 million cases of COVID-19 had been confirmed globally and more than 6.8 million people had lost their lives^12^. In Bangladesh, the total number of reported cases of COVID-19 infections was approximately 2.0 million by February 2023, which had resulted in more than 29 thousand confirmed deaths^12^.

Since its outbreak in late 2019, the pandemic has had a severe impact on society and regional healthcare systems around the world^3,13^ including the areas of reproductive, perinatal, and maternal health. In addition to the direct consequences of the infection itself, changes in healthcare systems, social policy, and social or economic circumstances have led to indirect consequences for reproductive and perinatal health in both high-income countries (HICs) and lower middle-income countries (LMICs), making women, in particular, vulnerable^14^. Domestic violence has reached a new peak^14,15^ and an increase in maternal mental health problems has been reported. An alarming increase in maternal mortality as an indirect consequence of the COVID-19 pandemic has also been estimated^16^. Another indirect consequence of the pandemic is the dramatic rise in demand for personal protective equipment (PPE) that has resulted in severe disruptions in the global supply chain and shortages of gloves, medical masks, face shields, gowns, and aprons have left frontline healthcare workers dangerously ill-equipped and unable to protect themselves and their patients from being infected with COVID-19 or other infectious diseases^17^. Due to the significant developments made in the midwifery profession in Bangladesh in recent years, it is of interest to identify any potential changes the pandemic has brought to existing barriers and the possibility of midwifery personnel to provide quality care. The aim of this study was to describe the possibility of midwives being able to provide quality midwifery care in Bangladesh during the COVID-19 pandemic, from the perspective of clinically active midwives, nurses in midwifery care, and midwifery educators.

## METHODS

### Design

Qualitative focus group discussions (FGDs) were conducted online, with clinically active midwives, nurses in midwifery care, and midwifery educators in four mixed groups. Qualitative thematic analysis inspired by Braun and Clarke^18^ was used to analyze the data. Ethical permission was provided by the Directorate General of Nursing and Midwifery in Bangladesh (2017).

### Recruitment and participants

To achieve a variety of perspectives, a purposive sampling method was applied. A total of 23 participants, comprising clinically active midwives, nurses in midwifery care, and midwifery educators were approached and asked to participate in the study. They were all undertaking a Master’s degree program in sexual and reproductive health and rights (SRHR) in Bangladesh, offered as a top-up education for midwives, nurse-midwives and midwifery educators by Dalarna University, Sweden, supported by UNFPA. The participants worked in different parts of the country, and in various types of healthcare facilities. They were all female and aged 39–47 years. Initially, the participants were contacted by email, to explain the objective of the research. The course supervisor then informed all the students in the program about the study, both orally during class and in writing through an online learning platform. Information given included an outline of the study and the voluntary nature of participation. Students were then contacted by phone (by NIP), had the study objectives explained to them, and were asked if they were interested in participating. All participants provided informed consent both over the phone and at the beginning of the recorded FGDs.

### Data collection

Data were collected through four internet-based FGDs^19^ with mixed participants of all three professional groups, in a virtual setting in Bangladesh. The criteria for selection were that all participants should be clinically active midwives, nurses active in midwifery care, or midwifery educators. All other healthcare professionals were excluded from the study. With a focus on the possibility of midwives being able to provide quality care in Bangladesh during the COVID-19 pandemic, an interview guide was developed to capture perceptions at individual, family, community, and societal levels^20^. The interview guide was created and pilot tested in an initial FGD. Only minor adjustments were needed, so it was deemed that these data could be included in the analysis. During the first part of each FGD, participants were asked to discuss threats and possibilities during the COVID-19 pandemic from the perspective of women’s lives and health in Bangladesh at individual, family, and community levels. The second part of the FGDs included questions addressing threats and possibilities for respectful care during labor and birth at the time of the COVID-19 pandemic on institutional and political levels. All FGDs were audio recorded. Each group met once, and the discussions were between 61 and 103 minutes in length. The FGDs were conducted by an anthropologist who was bilingual and an interpreter, and were held in English and Bangla, which was inspired by Chen and Boore^21^. Data were transcribed verbatim by the data collector (NIP) who translated all the parts spoken in Bangla into English during the process of transcription.

### Data analysis

Reflexive thematic analysis inspired by Braun and Clarke^18^ was used to analyze the collected data^18^. Phase one, ‘familiarizing yourself with the data’, started with two authors (JH, SG) reading the data individually several times to get a holistic understanding of the material. As the material was read, notes were made and ideas for coding were written in the margins. In phase two, ‘generating initial codes’, basic features of interest were identified and transformed into codes. The material and codes were placed in an excel document which became a basis for phase three, ‘searching for themes’. The excel document was used to organize codes, provide an overview, and create mind-maps with potential themes. During phase four, ‘reviewing themes’, codes, and data extracts were then reread, reanalyzed, and reorganized several times until patterns were established to form a clear and important context. During this process, similarities were noted with the domains of the analytical framework ‘What prevents quality midwifery care’ by Filby et al.^22^, a framework that has previously been used to explore quality midwifery care in Bangladesh^8,9^ , which led the initial inductive approach in a more deductive direction with the above framework applied as an analytical lens. The already created sub-themes with underlying codes and text extracts were reflected against the domains of the framework which thereafter in phase five, ‘defining and naming themes’, could guide the forming of main themes. Finally, appropriate names for the sub-themes were created. Citations from the data are used to illustrate results.

## RESULTS

The results comprise three main themes focusing on the possibility of midwives being able to provide quality care in Bangladesh during the COVID-19 pandemic: 1) Sociocultural perspectives with two subthemes; 2) Economic perspectives, with two subthemes; and 3) Professional perspectives, with three subthemes.

### Sociocultural perspectives

*Social views of COVID-19 jeopardize the security and health of midwives*

Beliefs in the community that midwives and other healthcare professionals carry the virus from hospitals to communities resulted in midwives developing ambivalent feelings towards their work. Being excluded and isolated from their families influenced the possibility of midwives being able to provide quality midwifery care at their workplace during the COVID-19 pandemic. Most of the interviewees received some degree of support from their own families, although some were not welcome to return home after work due to their family’s fear of COVID-19:

*‘Our relatives and neighbors treated us badly and saw what we were doing as a great sin when we helped COVID-19 patients in the hospital. They tortured us mentally and were aggressive. The pandemic era has left a scar on our minds and souls, it has also left us asking many questions, such as, to whom do we give care? Who are these people and why are they so aggressive towards us?’* (FGD4, P2)

*Lockdown reinforces gender inequalities and affects women’s health*

The possibility of midwives being able to provide quality midwifery care in Bangladesh during the pandemic was affected by women’s restricted movements during lockdown periods. Many women did not visit antenatal clinics for antenatal care or clinics for delivery care, which resulted in an increase of home deliveries. In addition, women did not visit clinics for family planning purposes resulting in an increase of unwanted pregnancies. Women were hence able to access midwifery care only in limited ways:

*‘Women who were pregnant were the worst affected because due to the pandemic they stopped visiting healthcare facilities for antenatal checkups. During lockdown periods women were not allowed to leave their homes and they didn't even visit the delivery centers to deliver their babies. This has been the major healthcare barrier.’* (FGD4, P3)

During the pandemic midwives also witnessed how frustration among men in society was often taken out on women. Women were victimized by their husbands and more women sought healthcare because of violence and rape:

*Since July (2020) the [monthly] number has increased ... All these are either gender-based violence or rape cases.’* (FGD4, P3)

### Economic perspectives

*Poverty in society aggravates midwifery practice*

Participants described a picture of increased poverty as a consequence of the pandemic as many people lost their jobs during lockdown periods. It was sometimes expected that the patients themselves should bury protection equipment. Thus, they continued to explain how unreasonable it was to expect patients to buy protective equipment such as masks to wear at the facilities, when they were already struggling to feed themselves and their families. This situation led to increased risk for midwives since they faced potential COVID-19 carriers who were presenting without PPE. This created high levels of stress among the midwives striving to provide quality care during the pandemic:

*‘We are caring for mothers who are part of outreach programs living in villages where they don't have access to healthcare and when they come to us they don't even have sandals/shoes to wear. How can we neglect them and how can we ask them to purchase safety equipment?’* (FGD1, P2)

### Professional perspectives

*Low professional status places midwives in challenging situations*

The possibility of midwives being able to provide quality midwifery care in Bangladesh during the pandemic was affected by their lack of influence. Participants expressed concerns regarding their low status and lack of influence as healthcare professionals. This was made visible in more explicit ways during the pandemic when their need for close communication with higher authorities and functioning structures was even more urgent. They felt that they were not listened to:

*‘Our voices are not reaching a higher level where things are being planned. No one is coming to listen to us. We are facing a huge barrier to communicate with higher authorities.’* (FGD2, P6)

The nurses, midwives, and midwifery educators were appointed to care for patients with symptoms of COVID-19 without adequate equipment, endangering themselves and the health of their patients, families, and communities. Women they were caring for, who were presenting with symptoms of COVID-19, were not always tested, and healthcare providers who were tested would stay at work until the test results came back, and sometimes they came back positive which spread the virus at the workplace:

*‘We are facing enormous difficulties in providing care to those who are not tested. We are in a dangerous position because we don't know if the patients we meet every day in the non-COVID-19 ward are infected or not. We are becoming more vulnerable and the risk of infection is high but the government is not prioritizing us at moment.’* (FDG3, P1)

*Deficient implementation of policies compromise safety*

Low rates of policy implementation, such as health packages and monetary help to health facilities and labor wards during the pandemic, were described. These safety measures had not reached the facilities of labor wards, which made it difficult for midwives to provide quality midwifery care without the fear of transmission of the virus and unreasonably long work shifts. The lack of access to safety measures, or ability to use them in a satisfactory way, plus long work shifts affected the possibility of midwives being able to provide quality midwifery care:

*‘During our shifts at the hospital, we can't follow the safety measures according to instructions. There is a difference between practice and theory. We are given a single set of PPE for a 12-hour shift. How scientific is it to wear a single set of PPE for twelve hours? I doubt its functionality and protectiveness.’* (FGD1, P3)

The greed of companies and individuals led to them taking advantage of society’s vulnerable position in order for them to make a profit. Selling fake safety equipment, for example, meant that women did not get the safe maternity care they deserved during the pandemic:

*‘There were many bad companies that started up during the emergency period, supplying low-quality or fake masks and equipment to the hospitals. Some of the employees and administrators of the health ministry were involved in these fake businesses. They made a syndicate to sell fake equipment to the hospitals and made a huge profit. These people made us suffer and put us [the health workers] in danger.’* (FGD4, P5)

*Increased workload for midwives endangers quality care*

The possibility of midwives being able to provide quality care was affected by an increased workload. Their field of responsibility was broadened due to the increase in severely ill patients, work outside the hospital, clinics reporting statistics regarding the pandemic to the health authorities, needing to attend home deliveries, and being available to women in the community by providing health advice over the telephone. While midwives worked in their spare time, lockdowns led to an increase in the number of women exposed to violence, unintended pregnancies, and abortions taking place in facilities:

*‘Many young girls were coming for treatment for unintended pregnancies, perhaps because due to the lockdown, the village 'quack' and homemade remedies were not available.’* (FGD 2, P3)

The considerable increase in workload included longer shifts, working overtime, and having little time for rest and recovery. This led to less time for proper sleep and midwives finding it difficult to maintain focus and provide quality care. The risk of infection during the pandemic made women with pregnancy-related complications wait at home for as long as possible, causing an increased number of emergencies. Some women went to private clinics first, because they believed the risk of infection was less there. Since private clinics experienced a lack of midwifery expertise, women would then present at general hospitals in a worsened condition, which added to the workload, and to the already existing lack of privacy. This, in turn, contributed to emergencies escalating even more, due to the care seeking delays.

*New possibilities for independent midwifery practice*

The COVID-19 pandemic opened up possibilities for midwives to practice midwifery care independently. It was described how midwives during the pandemic regularly provided intrapartum care to women without a physician’s involvement, as physicians tended to take a step back and work more at a distance from patients during the pandemic to avoid being infected. Physicians were seldom present in the labor ward; hence, midwives were left alone to manage the care provided, informing physicians by phone when possible. In this way, they worked more independently, with increased workloads and heavier responsibilities, but also with increased self-esteem, professional skill and confidence in providing quality midwifery care as a result:

*‘ ... So, this is a significant change that I pointed out – that we are providing all the services in the absence of the doctor, and we are becoming more skilled.’* (FGD1, P1)

## DISCUSSION

This study describes the perceptions of midwives, midwifery care nurses, and midwifery educators on midwives being able to provide quality care in Bangladesh during the COVID-19 pandemic. Widespread fear of the virus being transmitted, increased workload, and working in their free time affected the quality of care they provided. The COVID-19 pandemic reinforced preexisting barriers related to the newly established midwifery profession in Bangladesh on sociocultural, economic, and professional levels^8,9,22^, but also opened up possibilities for midwives to practice midwifery-led care independently.

The reported increase in workload due to the pandemic, with frustration as a consequence, confirms other studies describing a strained working situation and negative psychological impact on healthcare personnel during the COVID-19 pandemic^23-26^. During the pandemic, many midwives and nurses were assigned to care for patients with suspected or confirmed COVID-19 infections^25,27^. Their role as healthcare professionals has been vital in the fight against COVID-19^28^, but working on the frontline has not come without consequences. Studies show, in line with our findings, that the living situations of nurses and midwives worsened during the outbreak^27^ due to long working hours^28^, experiences of distress, burnout, isolation, and fear of contracting the disease^25,27,28^.

The lack of support midwives experienced from all areas of society, including from high-level officials and other cadres reported in our study, led to frustration and despair. This disrespect and neglect of midwives has previously been explained by the Filby et al.^22^ framework ‘What prevents quality midwifery care’ as a result of gender inequalities globally, and in Bangladesh^8,9^. The shortage of midwifery personnel, constituting a barrier for quality of care in Bangladesh, was reported prior to the pandemic^8,9^, partly due to the midwifery profession being newly established with still too few midwives being educated^8,9^. The link between shortage of staff and lack of quality midwifery care is not unique to Bangladesh, and has been described by Filby et al.^22^ as a concern worldwide. During the pandemic, with Bangladesh as example, these circumstances have, however, become more severe and visible. The combination of a heavy workload and the disrespect experienced has led to midwives experiencing difficulties in meeting demands, which has resulted in exhaustion^8,9^. Our findings thus shed further light on this already strained situation, and also on the dedication of midwives to serve the women they are assigned to care for. As the pandemic comes to an end, this is a resource well worth investigating further: ‘How can their confidence be strengthened at a structural level and in what ways can midwives increase their value and respect?’^22^. This is of great interest for sustained midwifery care in Bangladesh and the health of women and newborns in the long-term.

An increase in adequately educated and trained midwives is estimated to reduce the number of maternal, fetal, and neonatal deaths^2,29^ by up to 60% in a supportive working environment^2^. It has been described how the development of both the profession and care provided would benefit if midwives were given the possibility to practice independently, starting with midwifery students practicing the midwifery-led model of hands-on care, which is not limited by medical hierarchy^8-10^. Linked to this is also the finding suggesting how gender inequality and low professional status have a negative impact on quality midwifery care, which is in line with several other studies and the Filby et al.^22^ framework^8,9^. During the pandemic this was even more pronounced, exemplified in how midwifery personnel received less protective equipment than other personnel and how they were left to practice midwifery-led care independently during the pandemic. Working independently can have both positive and negative impacts on the quality of care provided, as midwives need to cooperate with other healthcare professionals in a well-integrated team in order to deliver high quality care when complications arise^2,9^. Midwives were left to work independently in the labor, birth, and postnatal wards during the pandemic, which may have showcased their ability to work independently with normal labors, births, and postnatal processes. It was actually possible to have an enabling environment in practice with doctors kept in the background but being reported to, and midwives performing vital tasks with good self-esteem, when the opportunity to do so was provided. This finding is supported by young Bangladeshi midwives who reported feeling comfortable in providing vital care to women under the constrained conditions seen in the Cox Bazaar refugee camps, while still pointing to the need for continuous training^30^. How midwives could be provided with an enabling working environment and utilized in the very best way possible, alongside the need for respect and teamwork within the health system, needs further investigation. An enabling environment, with midwives contributing towards the writing of healthcare policies, could have a positive impact on quality of care^2,31^, and the midwifery profession in Bangladesh could in the long-term implement a new model of care.

### Strengths and limitations

To minimize the risk of presenting misinterpreted results, the authors did reflect upon their experiences prior to analysis, and the primary analysis was conducted by the two authors who did not have any previous experience of the Bangladeshi context, thereby strengthening the credibility of the study^32^. Another factor strengthening its credibility was that the FGDs were conducted by a Bangla-speaking author, and recorded and transcribed verbatim, which reduced the risk of losing data due to misunderstandings, incomplete note taking or faulty recall^32^. Further, this made it possible to clarify uncertainties by replaying a sequence, if needed. This constitutes a strength in interpreting the data and presenting findings in an accurate way, thereby strengthening the dependability of the study^32^. The Bangla-speaking author could cross-check that the content shared in the FGDs was correct both during the interviews and in the transcripts. The FGDs comprised a mix of three cadres, based on the intention to achieve variety within the data. A side effect can, however, have been that a potential power tension between cadres may have silenced some perspectives. The online setting here may have limited the possibility to note such unspoken signals during the sessions. The qualitative design entails a limited number of participants, not allowing the findings to be generalized. The sampling method, participants, data collection, and analysis have been described in as much detail as possible to increase the transferability^32^.

## CONCLUSIONS

The pandemic jeopardized women’s safety and intensified gender inequalities, and midwives faced challenging circumstances due to their already demanding roles and low professional status. Gender inequalities, low professional status, lack of support, and heavy workloads threatening the health and safety of midwives – amplified during the pandemic – point to the need for respectful and dignified working conditions. The pandemic, however, also unveiled an opportunity for midwives to showcase their ability to practice independently, especially when it came to managing regular labors, births, and postnatal care. The willingness to implement a midwifery-led care model that is not yet used in Bangladesh, can in the aftermath of the COVID-19 pandemic be reinforced. Further research is suggested to investigate the medical safety of independent midwifery care in Bangladesh. A sustainable plan for educating new midwives, as independent care providers for normal pregnancy and intrapartum care, is vital for the contribution of midwives towards quality midwifery care in Bangladesh.

## Data Availability

The data supporting this research are available from the authors on reasonable request.
